# A Palpable Painless Axillary Mass as the Clinical Manifestation of Castleman's Disease in a Patient with Hepatitis C Disease

**DOI:** 10.1155/2016/1970276

**Published:** 2016-05-22

**Authors:** Athanasia K. Papazafiropoulou, Angeliki M. Angelidi, Antonis A. Kousoulis, Georgios Christofilidis, Chariklia Sagia, Liountmila Kaftanidou, Kassiani Manoloudaki, Aikaterini Tsavari, Georgios Kranidiotis, Alexandros Kamaratos, Andreas Melidonis

**Affiliations:** ^1^1st Department of Internal Medicine and Diabetes Center, Tzaneio General Hospital of Piraeus, 18536 Piraeus, Greece; ^2^Faculty of Epidemiology and Population Health, London School of Hygiene & Tropical Medicine, London WC1E 7HT, UK; ^3^Department of Pathology, Tzaneio General Hospital of Piraeus, 18536 Piraeus, Greece

## Abstract

*Introduction*. Castleman's disease (CD) is a rare lymphoproliferative disorder. CD is divided into two clinical subtypes: the most common unicentric and the less usual multicentric subtype. The majority of unicentric CD affects the mediastinum, while neck, abdomen, and axilla are less common locations.* Case Presentation*. Herein, we describe a rare case of unicentric CD in the right axilla in a 36-year-old white male with a medical history of hepatitis C virus infection admitted to our hospital due to palpation of a painless mass in the right axilla. Complete excision of the lesion was performed and, one year after the diagnosis, patient was free of the disease.* Conclusions*. Although infrequent, it is important to include CD in the differential diagnosis when evaluating axillary lymphadenopathy particularly in young patients with a low-grade inflammation process and chronic disease even in the absence of an abnormal blood picture or organomegaly.

## 1. Introduction

Castleman's disease (CD) or angiofollicular lymph node hyperplasia is a rare reactive lymphadenopathy of unknown etiology. Possible pathogenetic factors include follicular dendritic cell dysplasia and immune dysregulation [1]. CD can be divided into two clinical subtypes: the most common unicentric (or localized) and the less common multicentric. Localized disease is presented as lymphadenopathy and usually has a benign course [1]. On the other hand, multicentric disease has a worse prognosis, leading to death by malignancy [1]. CD can also be classified into two major histological variants: hyaline-vascular (HV-CD) and plasma cell variant (PV-CD). The HV-CD variant is more common than the PV-CD [2]. Of the 315 reported cases of unicentric CD in a French study, 65% were localized in the mediastinum, 16% in the neck, 12% in the abdomen, 3% in the axilla, and 4% in diverse locations [3]. Herein, we describe a rare case of unicentric CD in the right axilla in a male patient.

## 2. Case Presentation

A 36-year-old white male was admitted to our hospital due to palpation of a painless mass in the right axilla. The symptoms had presented for approximately a week. His past medical history included intravenous drug addiction (he had stopped use one year prior to admission) and hepatitis C virus (HCV) infection.

**Figure 1:**
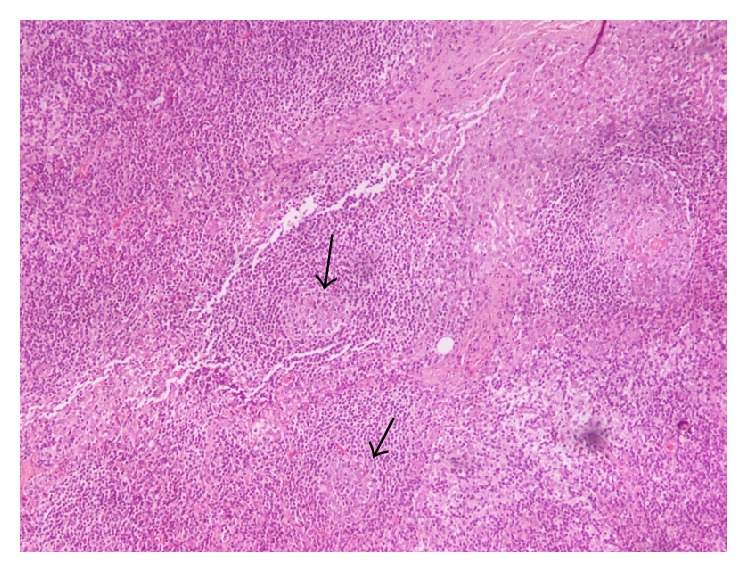
Preserved lymph node architecture. Residual follicles with atretic germinal centers.

**Figure 2 fig2:**
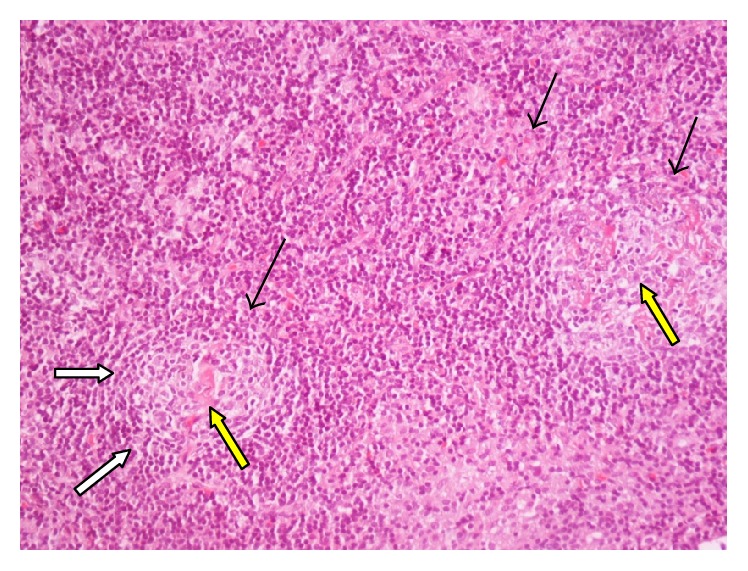
H-E regressed germinal centers (black arrows) surrounded by concentric layers of small lymphocytes (white arrows). Two follicles with hyaline-vascular changes (yellow arrow).

**Figure 3:**
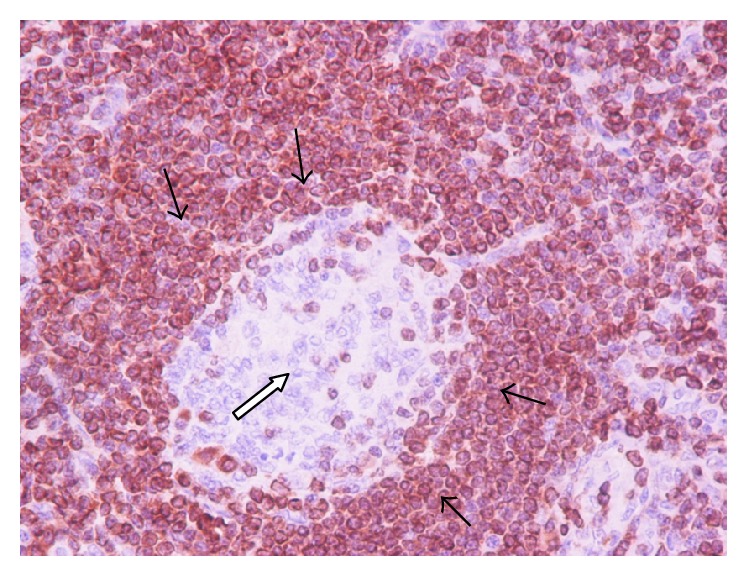
Lymphocytes in the mantle zone are positive (black arrows) for Bcl-2 and negative in the small regressed germinal center (white arrows).

Clinical examination revealed the presence of an enlarged lymph node group in the right axilla and multiple cat-scratches in his upper extremities. No other palpable lymph nodes were detected in the neck, the left axilla, and inguinal regions. Palpation of the abdomen revealed no tenderness or hepatosplenomegaly. Examination of his cardiovascular and respiratory system revealed no pathological signs. His vital signs were blood pressure of 120/80 mmHg, oxygen saturation of 99%, temperature of 36.6°C, and heart rate of 72 bpm. The remaining laboratory exams were within normal ranges. Computer tomography (CT) scans of the chest and upper and lower abdomen did not reveal any pathologically enlarged lymph nodes or other abnormalities. The initial differential diagnosis included (a) cat-scratch disease (given the contact of the patient with the cat), (b) other bacterial infections (staphylococcus and streptococcus), (c) viral infections (Epstein-Barr virus, cytomegalovirus, and human immunodeficiency virus (HIV)), and (d) neoplastic lymphohyperplastic disorders/lymphomas. Serological tests for* Bartonella henselae* (immunoglobulin G and M antibodies) were negative. His viral serology was positive for HCV while negative for HIV and herpesviruses. On the tenth day of hospitalization, a right axillary lymphadenectomy and a biopsy were performed. Histological examination revealed preservation of nodes architecture in the majority of tissue sample. Obliteration of subcapsular sinuses and regressed germinal centers in typically large follicles, some of which with “lollipop” features (onion skin appearance of the mantle zone lymphocytes and sclerotic blood vessels radially traversing into the germinal center), were detected (Figures 1 [Fig fig2]–3). Small number of plasma cells and immunoblasts were observed. These findings set the diagnosis of unicentric HV-CD. Patient had an uneventful postoperative course and he was dismissed a week later. One year after diagnosis, a throughout clinical examination and a CT scan were performed showing no evidence of remission of the disease.

## 3. Discussion

CD is a rare, benign condition that was first described in 1956 in patients with single mediastinal lymph node lymphadenopathy [1, 4]. The etiology and pathogenesis are unknown but an immunoregulatory abnormality is implicated in its development [1, 4]. An association of the multicentric type with human herpes virus- (HHV-) 8 infection in HIV-positive patients has been documented and plays an important role in the pathophysiology of the disease [5]. It seems that CD is the result of a chronic low-grade inflammatory process triggered by latent infection with HHV-8, which leads to lymphoid system hyperplasia and also stimulates secretion of interleukin-6 (IL-6) [6].

It has been suggested that CD lymph node abnormal overgrowth with or without systematic symptoms and organ dysfunction are probably caused by hypersecretion of cytokines. A proposed key pathogenic factor for CD is the elevated levels of circulating IL-6 [7].

Thus, of interest in this case, while the patient was negative for HHV-8 and HIV infections, his serological tests for HCV were found positive. Studies have documented the elevated levels of IL-6 in HCV infection [8, 9].

However, to the best of our knowledge, there has been no association between HCV infection and CD documented. Extremely few case reports of coexistence of CD (especially the multicentric type of disease) with HCV infection have been published [10–13]. In our case, a potential relationship of HCV-induced elevated IL-6 levels and the development of the CD cannot be supported while further investigations and evidence are needed.

Unicentric CD is usually presented as mediastinal, hilar, or intra-abdominal lymphadenopathy [1, 2]. Primary axillary localization of HV-CD (as in our patient) accounts only for 2-3% of the cases [14–17]. CD affects predominantly young adult patients without gender predominance [2]. Patients are presented with localized mass often detected accidentally or with general symptoms such as fever, night sweats, and weight loss. Symptoms related to compression of adjacent tissues are rarely detected [2].

The most commonly described (77–91%) symptom of unicentric CD is a mass (usually painless), like in our patient [2]. Common laboratory anomalies include anemia, hypoalbuminemia, polyclonal gammopathy, elevated erythrocyte sedimentation rate or C-reactive protein concentration, and proteinuria [18]. In the present case, patient's laboratory exams were normal. Diagnostic imaging methods such as ultrasound and CT or magnet resonance imaging were not specific. Hence, the gold standard for diagnosis is pathological examination [19].

Treatment of choice for unicentric HV-CD is complete resection, with excellent long-term prognosis (5-year survival rate of nearly 100%) and uncommon relapse [1, 20]. However, patients may develop malignant neoplasms, such as follicular dendritic cell sarcomas or vascular neoplasms, associated with HV-CD [20]. Radiotherapy has also been reported to be effective in some patients with a variable response rate and usually administered to alienate compressive symptoms [21].

## 4. Conclusions

In conclusion, primary axillary localization of CD is a rare but possible cause of peripheral lymphadenopathy. Although infrequent, it is important to include CD in the differential diagnosis when evaluating axillary lymphadenopathy particularly in young patients with a low-grade inflammation process and chronic disease even in the absence of an abnormal blood picture or organomegaly.
